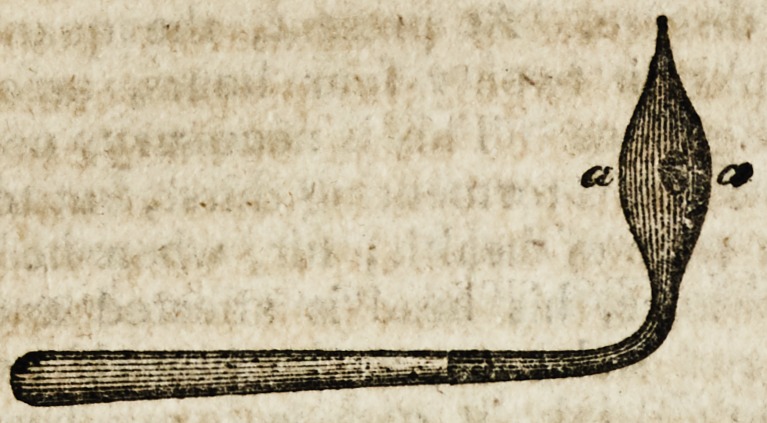# An Enquiry into the Circumstances Which, in the Application of the Ligature to Arteries, Are Capable of Preventing the Success of Its Operations, by the Production of Hæmorrhage

**Published:** 1816-03

**Authors:** 


*For the London Medical and Physical Journal.*

### *An Enquiry into the Circumstances which, in the Application of the Ligature to Arteries, are capable of preventing the Success of its Operations, by the Production of Hæmorrhage;* by E. C. B.

**Quot homines! tot sententiæ sua cuique vera!**

COMPARED to the degree of alarm which was formerly occasioned, not only on the part of the patient, but also of the surgeon, from the accidental occurrence of hæmorrhage, that now experienced by either, from the influence of similar circumstances, is comparatively insignificant. It cannot for a moment be conceived, that hæmorrhage arising from accidents was an occurrence which less frequently happened at that than it does at the present period, and thus enable us to account for the premised fact, by supposing that, in consequence of the rarity of wounded vessels, the terror of the ancients was increased by the novelty of that alarming circumstance. This will undoubtedly be deemed unworthy of a consideration; and the material advance which the profession has experienced on this head can only be attributed to the improvements which have been made on the former modes of stopping hæmorrhage. For the accomplishment of this desirable effect, it was formerly the custom to make use of styptics and cauteries, capable of producing the most violent operations; and, on failure of these, to employ, without reserve, a heated iron to the whole bleeding surface: these, for a time, by producing a mortification of the part, were not unfrequently successful in affording a temporary cessation of this evacuation; but the events which were subsequently produced by the separation of the sloughs, the inevitable consequence of these operations, upon the view which we now take of this subject, may be seen detailed in every page of such works as possess a faithful relation of cases in which such means were had recourse to.

Notwithstanding the daily occurrence of cases which thus met with a fatal termination, that remedy which is now employed ployed with such unlimited success for the restraint of hæmorrhage, and the adoption of which has constituted the entire origin of all our most recent innovations and improvements in the art of operative surgery,—the adoption of which now enables the medical attendant to behold with calmness his patient, although suffering from the wound of even one of the larger arterial trunks, which has rendered the operation of amputation simple, the recovery of the patient after such an operation more capable of anticipation, and the adoption of which has caused such an operation to become unnecessary, in the cure of aneurism of arteries supplying the extremities, and has occasioned this horrid mutilation to be avoided, by the substitution of an operation but of small importance. The remedy which is capable of being so advantageously productive consists in the ligature; and, surprising as it may appear, this was insignificantly included among the numerous remedies which the ancients were accustomed to employ for the securement of bleeding vessels.

The invention of the ligature has hitherto been ascribed to the ingenuity of Ambrose Paré, and moderns are lavish in their praises of this author for having become so materially useful to the chirurgic art. The modesty with which this ingenious gentleman relates the advantages of its application, may be seen by referring to his voluminous work on this subject, wherein he seems convinced that this discovery was not the product of his talents for ingenuity, but that he became acquainted with its use by a sort of inspiration. Inspirations of this sort are by no means uncommon: we not unfrequently see starting up as new that theory or invention which was, perhaps, old and obsolete a hundred years ago; and less unfrequently do we find one author engaged in stripping the laurels from the brow of him on whom justice has bestowed them, and disgracefully attempt to appropriate to himself these *spolia minervæ*. The fact is this, Ambrose Parè is as little entitled to the merits which are due to the inventor of the ligature, as is Dr. Jenner to the discovery of vaccination; to both of whom are due praises of no inferior kind, as the promulgators of practices to the adoption of which the whole world will ever be indebted. If we take the trouble of perusing chap. 26, book v. of Celsus, entitled "Curatio adversus profusionem sanguinis in vulneribus," we shall find, that, after mentioning the other usual and then superior remedies for the restraint of hæmorrhage, the following observation is carelessly made, which is sufficient to overthrow all claims which may be made by Parè to the *invention* of the ligature. "Quod si illa illa quoque profluvio vincuntur; venæ, quæ sanguinem fundunt, apprehendendæ circaque id, quod ictum est, duobus locis deligandæ, intercidendæque sunt, ut et in se ipsæ coeant, et nihilominus ora præclusa habeant." From this passage it is apparent that to Ambrose Parè the profession is not indebted as the inventor of the ligature, though materially so as the first author who deservedly esteemed its application, and published to the world its advantages over all other remedies.

At the time of this important introduction into the art of surgery, and when first, by the work of Parè, it was presented to the public eye, it was not, as may be expected, so eagerly embraced by those in the habit of performing operations, but met with a considerable degree of opposition by the generality of surgeons, who valued their remedies in proportion to the cruelty of their application, and who, on this account, preferred the torturing custom of cauterizing the bleeding vessel either by powerful chemical agents, or, in some case, the actual cautery, to the mild operations of the ligature. Another cause, which, without doubt, materially attributed to the opposition which the adoption of the ligature experienced, was the unsuccessful termination of innumerable cases in which it had been used, and which, we are recently taught, was assignable to the ignorance which then prevailed with respect to the modus operandi of its application, in securing the orifice of the bleeding vessel. At that time it was the practice to detach the vessel from all its communications, and, what was then supposed prudent, to introduce betwixt the ligature and artery some soft substance, so as to prevent the laceration of either of the arterial coats. The former of these practices, we are now convinced, by thus insulating the artery, and depriving it of all vascular communication, cannot fail of being productive of secondary hæmorrhage, by causing the mortification, and consequent detachment, of the arterial parietes above that part to which the ligature was applied; and the latter precaution, we are now aware, was calculated to prevent the very object, by the observance of which the ligature is efficient in restraining hæmorrhage. Be it then to the honour of Dr. Jones, who first ascertained these practical desiderata, and who, by the accurate performance of experiments, also obtained a knowledge of the whole modus operandi of the ligature, and since whose remarks this valuable application has not only been universally adopted, but the success which has attended that adoption has been most materially advanced.

When a ligature is placed upon an artery, the first of its effects effects is the formation of a coagulum in that part of the vessel which is nearer to the heart, and this of itself would be capable of partially resisting the flow of blood, but is immediately useful in preventing the impetus of the blood's motion falling wholly on that part of the artery encompassed by the ligature: this, however, is only an assistant appointed by Nature to the accomplishment of one great effect, viz. the complete obliteration of the arterial tube. By the application of a ligature, and to promote the successful event of its operations, it is actually necessary that the internal coat of the artery be divided, and, by that division, as vessels must inevitably be wounded, so they, in like manner with other wounded vessels, throw out coagulable lymph. As the effect of the ligature is to retain in contact the opposite divided edges of this arterial coat, by this effusion they become agglutinated, and gradually as the remaining portion of the parietes of the vessel becomes ulcerated by the pressure of the ligature, that also becomes united by the same process; and previous to the detachment of the ligature, the artery is completely divided. These are the facts which the experiments of Dr. Jones have been efficient in establishing, and from which may be derived every practical information that can be deemed necessary. But notwithstanding so much has been said on this subject, not only by the abovementioned, but by several more recent authors, hæmorrhage subsequent to the application of ligatures on arteries is by no means an accident extinct from occurrence even at the present period: an enquiry, therefore, into the causes of such failure cannot be considered intrusive on your valuable pages.

The objects to be aimed at in the application of a ligature on an artery are as follows:

The passage of the ligature around its parietes, without detaching, in the smallest degree beyond the necessary extent, its external coat from the enveloping substance which surrounds it, and through the medium of which the proper tunics of the artery are supplied with blood. When this is accomplished, the ligature is to be tied with such a degree of firmness as is sufficient for the production of the division of the internal coat of the artery. Another and very important object to be obtained is the non-inclusion of any other substance, and allowing only the artery itself to fall within the grasp of the ligature.

These are the three important points to be observed in the use of the ligature, and the neglect of either one of which is sufficient in itself of producing all the ills arising from the accidental occurrence of secondary hæmorrhage.

The first, viz. the passage of the ligature around the parietes of the artery, without (in an extent beyond that which is absolutely necessary for its accomplishment) effecting its detachment from the enveloping substance by which it is supplied with blood, becomes advisable in compliance with the following facts. No artery is capable of supplying its own parietes with blood, but they are always supplied from a neighbouring artery, through the medium of the enveloping membrane by which they are encompassed: this fact was first ascertained by Mr. Hunter, who, without avail, endeavoured to inject the vasa vasorum of different arteries, by pouring mercury into their tubes. If, therefore, through the medium of any substance, an artery be supplied with blood, the consequences of the detachment of that substance from its external tunic, to the eyes of every person, must be obvious. We know that when the circulation through any part (which, in a natural state, is in a state of organization) ceases, mortification is the inevitable occurrence: we are also aware that the detachment of the mortified portion by the process of absorption is as inevitably the consequence of the mortification; and few there are who are not apprised that, by the detachment of any portion of the tunics of an artery in the calibre of which blood continues to circulate, hæmorrhage must be the effect.

The second becomes a very important object in the operation; for the division of the internal tunic of the artery is the *sine quâ non* of its successful termination. The division of the internal coat of the artery becomes a necessary precaution, for the same reasons that the ulcerated edges of a wound are not as capable of becoming as speedily united as those of one more recently inflicted by a sharp instrument—by the division of this coat at the time of applying the ligature, the vasa vasorum by which it receives its supply of blood are wounded, and thus, in compliance with the law which influences other vessels in the same situation, they pour out coagulable lymph. As the ligature is so applied as to make constant and equal pressure on the artery, these divided coats are retained in contact, and, in a similar manner to other recently-divided parts, become united by the first intention. If, on the other hand, the internal tunic of the artery be not, at the time of its application, divided, its division must ultimately take place by the ulcerative process occasioned by the constant pressure of the ligature; and, in this case, the ligature, instead of being effectual in retaining in contact the edges of the coat recently divided, its powers are ill bestowed, and by them the two ulcerated surfaces of arterial pariety are approximated, and the chance of union wholly destroyed; destroyed; when, therefore, the ligature, by its pressure, has ulcerated its way through the artery, it becomes detached, and, as from the above cause no union of the edges of the arteries can possibly have taken place, hæmorrhage must ensue. Another circumstance, in the neglect of which secondary hæmorrhage must be the consequence, is the non-inclusion of any substance surrounding the artery in the embrace of the ligature. If a piece of muscle be included together with an artery in a ligature, although the diameter of the former shall materially exceed that of the latter, (if an appropriate degree of force be made use of in the application of this ligature), it is very probable that the innermost coat of the artery may be divided, and the operation so far rendered complete; for, if a piece of muscle, exactly twice the diameter of an artery, be included in a ligature, if the capability of resistance of the former doubly exceed that of the latter, an equivalency is observable, and the innermost arterial tunic will be cut prior to the inflection of any laceration on the muscle; it is not then from this cause, that, where a substance is included, together with an artery in a ligature, the unsuccessful termination of the operation is unavoidable. When, together with the artery intended to be secured, any portion of muscle or other substance be included, we will suppose (as is very probably the case,) that the innermost tunic of the artery is divided, in consequence of its inferior capability of resistance compared with that which the muscle possesses; but, as I have ascertained by experiment, although this is the case with respect to the internal coat of the artery, its external coats are capable of offering as much or even more resistance than the muscle which is included.

I included in a ligature, together with a portion of muscle, a part of the femoral artery of a subject actually dead: the diameter of the muscle three times exceeded that of the parietes of the artery, yet a small degree of force was only necessary to accomplish the division of the internal coat, such as was not sufficient to produce any laceration of the muscle itself. I now applied the same piece of muscle to another portion of the same artery, and with a similar degree of force effected the division of the internal, but no degree of force, inferior to that which was capable of dividing the muscular substance, was competent to the division of the external coats.

This experiment I have frequently repeated with little variety of result, and it is calculated to prove, that the muscular structure is capable of thrice the resistance the internal coat of the artery is enabled to exercise, whilst the degree of resistance which is capable of being made by the external coats three times exceeds that of the internal, and is equivalent to the muscle itself. This experiment I have here introduced for the purpose of ascertaining by what process the inclusion of a portion of muscle or any other substance, is effectual in being productive of secondary hæmorrhage, and which is as follows:—

Allowing that by the application of a ligature, under such circumstances, (notwithstanding the intervention of some substance betwixt it and the external coat of the artery) its internal is divided prior to the laceration of the intervening substance, and the operation therefore not rendered incomplete on that account; if, as this experiment proves, the external coat is capable of making a resistance equal to that of the intermediate muscle, although this shall have taken place, the ulceration which the pressure of the ligature must unavoidably make, and by which its detachment must ultimately be produced, instead of beginning from within, and thus, previous to its detachment from the artery, causing its edges to unite, the capability of resistance being equal betwixt the external coats and muscle, it necessarily begins externally in the latter, and, as it proceeds, the ligature becomes loosened; till at last all pressure from the artery itself is secured, and hæmorrhage cannot fail to be the result.

The detachment of the artery from its surrounding substance to an unnecessary extent; secondly, the non-division, by its application of the internal coat; and, thirdly, the inclusion of a portion of muscle or other substance, together with the artery in a ligature,—are certainly the three principal causes of the frequent unsuccessful terminations of those cases in which the ligature has been applied. But, notwithstanding a due observance to the prevention of the occurrence of hæmorrhage from either of these causes has been rigidly enforced, and that by most skilful operators, in making use of the ligature; it will sometimes happen, that on or before its detachment, hæmorrhage will ensue. From what cause does it then happen? The presence of ossific matter in that portion of the arterial coats immediately in the embrace of the ligature, has, by some authors, been supposed not unfrequently to be the cause of secondary hæmorrhage, by preventing the union of the sides of the vessel; and, however plausible this may appear, we have undoubtedly facts on record in which such has not been considered objectionable to the application of the ligature, and no hæmorrhage has taken place; but, notwithstanding these are mentioned by creditable authors, the application of a ligature on a portion of artery thus diseased, cannot but render unsafe unsafe the operation, and should always (if it is possible) be avoided on that account.

A certain degree of inflammation is always necessary to effect the union of two edges of a wound, and, in conformity to this law, a certain quantity is requisitely present during the union of the two divided portions of the internal coat of an artery, when a ligature has been applied, and, by the application of which, this inflammation is produced. If this degree of inflammation be nearly obtained, so as to suffer no deviation either from excess or deficiency, the agglutination of the arterial parietes will be effected; but it must be understood, that although by inflammation to this extent, the sides of the artery are united; if it is permitted to exceed this extent, the very contrary will take place. Whilst inflammation to this is existent, it is termed adhesive; but, if it be permitted to exceed this extent, instead of the adhesive, the suppurative or ulcerative stage of inflammation begins its ravages, and the consequences of ulceration in the present instance must be evident. Subsequent to the application of a ligature upon an artery, all inflammatory action in the constitution at large should be most carefully and speedily reduced, since the small degree of inflammation which is requisitely excited in the artery itself cannot be expected at all to affect the system, although capable of suffering a degree of diminution from constitutional treatment; and bleeding, if necessary, should not be, on this account, avoided. The consequence of allowing the extent of the inflammation to exceed that which is requisite for the promotion of the adhesion of the arterial parietes, and thus allowing the ulcerative or suppurative stage to commence is, without doubt, sometimes the cause of hæmorrhage, and the deficiency of inflammation to the necessary extent, from an indolency of habit in the patient, may be productive of the same effect; but there still exists another cause to which this accident, I am persuaded, may be, in the greater number of instances, attributed.

The number of accidents of this kind, which take place subsequently to the operations of amputation, (notwithstanding the plurality of arteries to be by the ligature secured in this case, and the unfavourable circumstances under which this operation is sometimes undertaken,) is not more than once to every third time, proportionally, that hæmorrhage follows the application of ligatures on arteries in the operation for the cure of aneurisms. Did this accident arise from the unnecessary detachment of the artery, from the non-division of its internal coat, from the inclusion of a portion of of muscle, together with the artery in the ligature, from the presence of its ossific nature in the parietes of the vessel, from a deficiency of inflammation not constituting the adhesive, or from an excess of inflammatory action extending it to the suppurative stage? the proportionate number of cases would be equal, since either of these causes would be equally capable of taking place in cases of amputation as in those in which arteries are tied for the cure of aneurism. It is clear, therefore, that hæmorrhage sometimes follows the application of ligatures on arteries to the latter effect, independent of the operations of either of the above-mentioned causes.

The only deviation which can possibly be made, with respect to the artery in either of these operations, consists in the method by which the ligature is conveyed around the artery to be secured, and it is therefore reasonable to suppose that it is in some accidental circumstance which takes place in this conveyance which is productive of so alarming an event.

The mode in which ligatures are applied to bleeding arteries after amputation, cannot but be known to every one of the medical profession; and the only precaution which is necessary to be observed, is the non-detachment of its coats by extending it too much by the forceps; but, as the artery will suffer a considerable degree of extension without denuding its external coat from the cellular vascular substance which surrounds it, in consequence of the elasticity which the latter is capable of exercising, this, even, is seldom capable of producing subsequent inconvenience in the occurrence of hæmorrhage. When, however, the femoral artery, or any other which is necessary to be tied for the cure of aneurism, is about to have a ligature passed around it, this cannot be done without an instrument, and here we arrive at the cause of all the mischief. The instruments which are constructed for this purpose, and which are in pretty general use, are termed aneurismal needles; they are of different sizes and shapes, but the greater number of them calculated to be productive of the most serious consequences. As every, even the smallest, portion of an artery is liable to become disorganized and in a state of mortification, from the denudation of its coats, it would seem advisable never to employ any instrument which exceeded in size the ligature itself, to effect its passage around the artery; since, in this case, no part of the vessel could be denuded but that which was immediately encompassed by the ligature, and, consequently, no part could become in the above state, excepting that, that, the ulceration of which is a desideratum. On the other hand, aneurismal needles of this shape are not uncommon.

The distance, which in general is observed betwixt *a* and *a*, is, on a moderate scale, not less than three-quarters of an inch, whilst, from the numerous advantages which accrue from the application of extremely fine ligatures, the ligature does not exceed in diameter one-twentieth of the same space; consequently, three-quarters of an inch of the artery is denuded, whilst the ligature in the mid-space embraces only one-twentieth part of that which is denuded; and, therefore, the artery is detached to twenty times the extent which is necessary for completion of the operation, though not more than is sufficient to admit of the passage of this awkward instrument. There are many other contrivances for the passage of a ligature around an artery, some of which I confess, are not so well calculated to produce a mortification of a portion of the coats of the vessel and the subsequent hæmorrhage; but, whilst there are many adapted less artfully to this purpose, I may, with safety say, there are others of much worse construction.

*January* 28, 1816.

## Figures and Tables

**Figure f1:**